# CAV1 as a Potential Regulator of Endothelial–Macrophage Communication Through the CD74/MIF Pathway in Breast Cancer

**DOI:** 10.3390/ijms27209298

**Published:** 2026-10-20

**Authors:** Liangshuang Li, Kaiyuan Xing, Shuang Feng, Zhen Wang, Lingling Zou, Haotian Guo, Jiang Zhu

**Affiliations:** 1 School of Medical Informatics, Daqing Campus, Harbin Medical University, Daqing 163319, China; 2 Key Laboratory of Frigid Zone Exercise Health Research and Translation in Heilongjiang Province, Xinyang Street 39, Daqing 163319, China

**Keywords:** breast cancer, CAV1, endothelial cells, scRNA-seq, cell–cell communication

## Abstract

Breast cancer (BRCA) is one of the most common malignancies and a leading cause of cancer-related mortality in women. We integrated predicted compound targets of Fufang Banmao Capsules with BRCA-associated genes and prioritized candidates using LASSO regression, correlation analysis, and ROC analysis. Single-cell RNA sequencing analysis then characterized their expression patterns, endothelial heterogeneity, and inferred endothelial–macrophage communication. The four key genes CAV1, CES1, PPARG, and RUNX1T1 showed the highest AUCell scores in endothelial cells. Cell–cell communication analysis inferred MIF signaling involving CD74-containing receptor complexes between endothelial cells and macrophages. Virtual knockout analysis identified CD74 among the genes predicted to be perturbed following CAV1, CES1, or RUNX1T1 knockout in endothelial cells. Further analysis revealed heterogeneous CAV1 expression across endothelial subpopulations, with relatively higher expression in ACKR1-positive and metabolic endothelial cells. Pseudotime analysis characterized variation in CAV1 expression along inferred trajectories, while regulon analysis identified distinct regulatory programs across endothelial subpopulations. Together, these findings prioritize candidate genes linked to the predicted targets of Fufang Banmao Capsules and generate hypotheses concerning their cellular context in BRCA.

## 1. Introduction

Breast cancer (BRCA) is a highly prevalent malignant tumor among women and ranks as one of the three most common cancers worldwide, along with lung and colorectal cancer [1,2]. In recent years, the incidence of BRCA has been steadily increasing due to environmental changes, lifestyle shifts, and population aging, among other factors [3]. BRCA not only seriously impacts patients’ health, leading to breast lesions and impaired body function, but also poses a significant threat to survival rates [4]. Numerous studies have demonstrated that early detection of BRCA can substantially improve the five-year survival rate. For example, after receiving standardized treatment, the five-year survival rate of patients with early-stage BRCA can reach over 90%, while that of patients with advanced-stage breast cancer drops significantly to 20–30% [5]. In our country, BRCA has emerged as the most frequently diagnosed malignant tumor among women, with the age of onset gradually decreasing. Magnetic resonance imaging (MRI) can aid in differentiating benign from malignant breast lesions [6]. Serum tumor markers, including CA 15-3 and CEA, may complement clinical and imaging assessments when monitoring treatment response in patients with metastatic breast cancer [7]. The main treatments for BRCA are chemotherapy, radiotherapy, targeted therapy, immunotherapy, and endocrine therapy.

However, these treatments have limitations; taxane-based chemotherapy for breast cancer can cause clinically significant toxicities such as neutropenia and peripheral neuropathy [8]; radiotherapy can harm peripheral tissues; immunotherapy has immune-related reactions. Given these challenges, it is important to explore novel therapeutic strategies to improve the patient’s condition. Due to its multi-component and multi-target characteristics with relatively low toxicity, traditional Chinese medicine (TCM) has attracted increasing attention as a complementary and alternative therapeutic approach for cancer treatment in this context. Fufang Banmao Capsules, a traditional Chinese patent medicine, have been widely used in cancer treatment, with Banmao as their primary anticancer ingredient. Other components include Sanleng, Ginseng, Huang Qi, and several other herbs, among which β-elemene from Sanleng has been reported to interfere with tumor cell cycle progression.

In recent years, single-cell RNA sequencing (scRNA-seq) and transcriptomics technologies have been integrated to gain comprehensive insights into the initiation and progression of BRCA. In this study, we employed transcriptomic and single-cell analyses to identify target genes, core biological processes, and major signaling pathways associated with BRCA. Additionally, the potential correlation between the target genes and infiltrating immune cells was explored in depth by single-sample gene set enrichment analysis (ssGSEA). At the single-cell level, scRNA-seq data were used for virtual knockout to dissect the specific cell types significantly affected by the target genes, and to observe the expression changes in key genes, offering a deeper understanding of the anti-BRCA mode of action of Fufang Banmao Capsules at the cellular level.

In summary, this study systematically investigated the mechanism of action and potential targets of Fufang Banmao Capsules in BRCA treatment through transcriptomic and single-cell analyses. These findings not only elucidate the potential action pathway of Fufang Banmao Capsules but also lay a solid theoretical foundation for its clinical application and further research.

## 2. Result

### 2.1. Breast Cancer Disease and Target Genes Associated with Fufang Banmao Capsules

**Figure 1:**
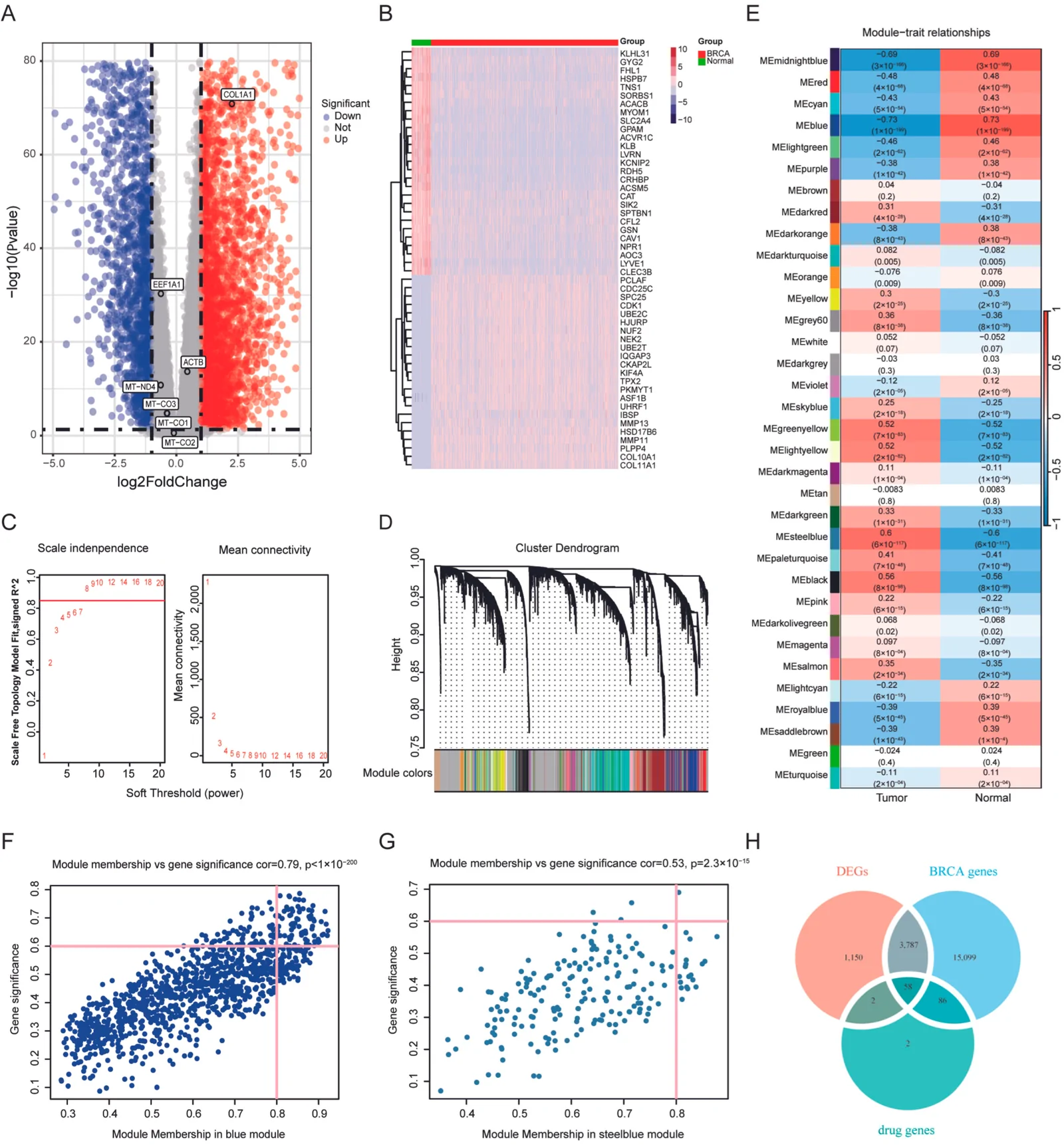
Identification of candidate genes. (**A**) Volcano plot displaying DEGs between BRCA and control samples. Red and purpl dots represent significantly upregulated and downregulated genes, respectively (|log2FC| > 1, adjusted *p*-value < 0.05). (**B**) Hierarchical clustered heatmap of the top 50 most significant DEGs, ranked by adjusted *p*-value. (**C**) Scale-free topology model fits index and means connectivity for determining the optimal soft-thresholding power (β = 8). The dashed line indicates the scale-free topology fit (R^2^ > 0.85). (**D**) Clustering dendrogram of genes based on the measurement of dissimilarity. Different modules are represented by different colors. (**E**) Module–trait relationship analysis. The horizontal axis represents sample traits, and the vertical axis represents co-expression modules. Color intensity indicates the correlation strength between modules and traits. (**F**,**G**) Module–trait relationship analysis diagram of hub modules. Correlation plots depicting GS-MM relationships for BRCA in steelblue and blue modules. (**H**) Venn diagram illustrating the intersection of DEGs, drug genes, and BRCA genes.

To identify disease-related targets for BRCA, we first retrieved BRCA-related disease targets from the GeneCards and OMIM databases, resulting in a combined set of 18,739 genes (Supplementary Table S1). Next, differentially expressed genes (DEGs) were performed using the R package “DESeq2” [9] to compare BRCA samples with control. We obtained 4997 DEGs, including 3039 upregulated genes and 1958 downregulated genes (|log2FC| > 1 and adjusted *p*-value < 0.05). A volcano map was constructed using the ggplot2 package to visualize the DEGs (Figure 1A), followed by a hierarchical clustered heatmaps of the top 50 most significant DEGs (ranked by adjusted *p*-value) using the ComplexHeatmap package [10] (Figure 1B). Subsequently, we constructed a weighted gene co-expression network analysis (WGCNA) and grouped genes with highly coordinated expression into a single gene module. After removing 19 outlier samples using the hclust function, 1178 samples were retained for subsequent analysis (Supplementary Figure S1A,B). The excluded samples comprised 18 BRCA samples and one normal sample. No statistically significant difference in sample-type distribution was detected between excluded and retained samples (*p* = 1.000). Among BRCA samples with available staging information, tumor stage distributions also did not differ significantly between the two groups (*p* = 0.2097; Supplementary Table S2). Scale-free topology fitting determined an optimal soft-thresholding power of 8 (fitting index R^2^ > 0.85, mean connectivity close to 0; Figure 1C). These genes were classified into 35 co-expression modules (Figure 1D). To prioritize modules associated with BRCA status, we compared module eigengene–trait correlations across all modules. The blue module exhibited the strongest negative correlation with tumor status (r = −0.73, *p* = 1 × 10^−199^), whereas the steelblue module showed the strongest positive correlation (r = 0.61, *p* = 6 × 10^−117^; Figure 1E). These two modules were therefore prioritized to represent the strongest disease-associated co-expression patterns in each direction. Together, the blue and steelblue modules contained 1261 genes, which were retained as candidate BRCA-associated genes (Supplementary Table S1). Correlations between module membership (MM) and gene significance (GS) were observed in the blue (r = 0.79, *p* < 1 × 10^−200^) and steelblue (r = 0.53, *p* = 2.3 × 10^−15^) modules (Figure 1F,G). Combining these genes with 18,739 database-derived BRCA-associated genes yielded 19,030 unique candidate BRCA-associated genes after deduplication. We combined the disease-associated genes obtained from hub modules and the 18,739 disease genes obtained from the database download as BRCA-associated genes, yielding a total of 19,030 disease-associated genes. The intersection of DEGs, drug target genes, and BRCA-associated genes was analyzed using the VennDiagram package [11], yielding 58 candidate genes for subsequent analysis (Supplementary Table S3).

### 2.2. Functional Enrichment Analysis of Candidate Genes

**Figure 2:**
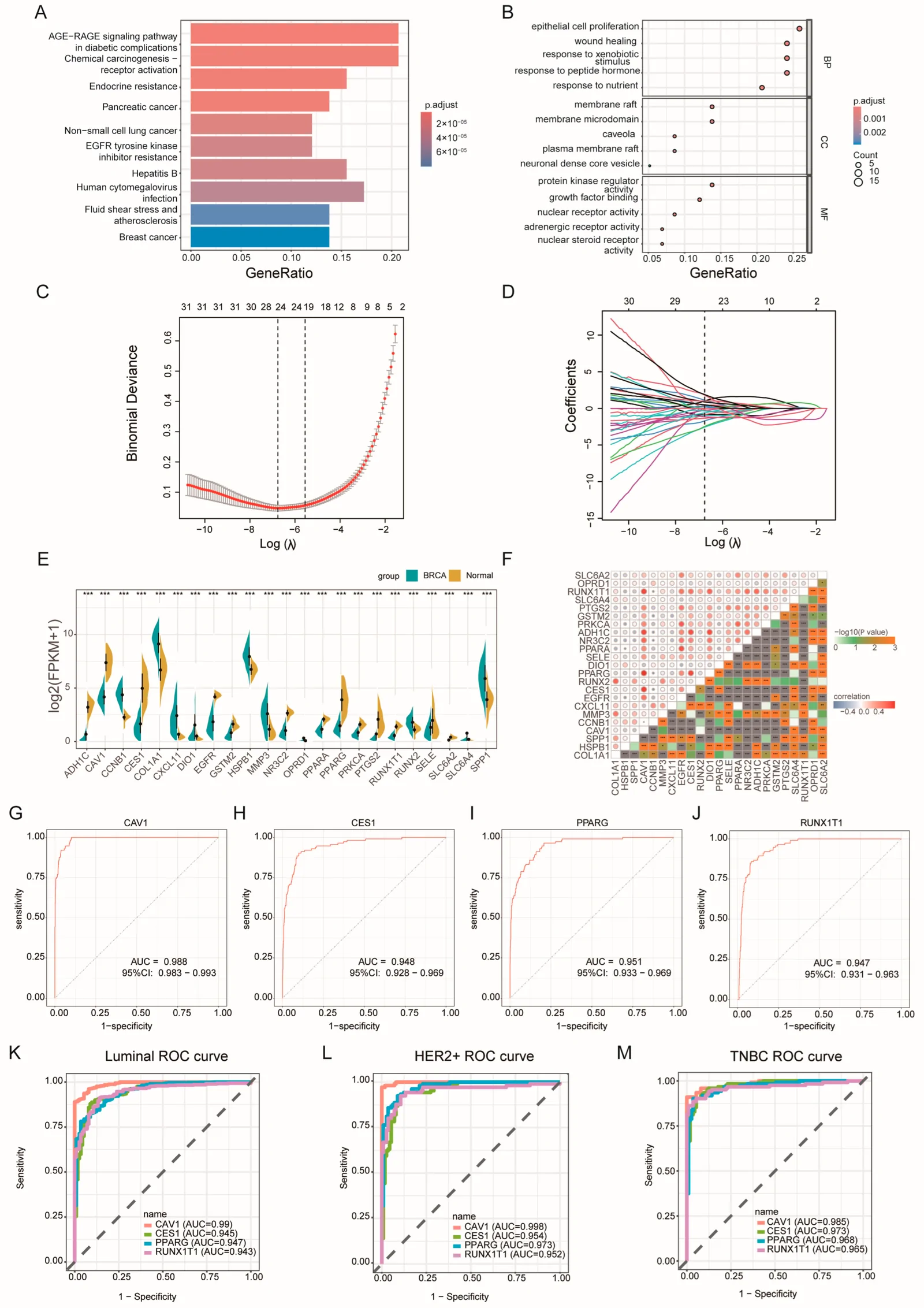
Functional enrichment. (**A**,**B**) GO enrichment results, including BP, CC, and MF. KEGG pathway enrichment results. (**C**) LASSO regression analysis for gene selection. Binomial deviance versus log(λ) plot, showing the optimal λ value for gene selection. (**D**) Coefficients versus log(λ) plot, illustrating the shrinkage of coefficients as λ increases. Each colored line represents a distinct gene, showing how its regression coefficient changes with Log(λ). (**E**) Comparison of the expression levels of the target genes between the control and BRCA samples. Significant differential expressions are highlighted (* *p* < 0.05, ** *p* < 0.01, *** *p* < 0.001). (**F**) Pearson correlation analysis of target genes, demonstrating the relationships among the 23 selected target genes. (**G**–**J**) ROC curves of CAV1, CES1, PPARG, and RUNX1T1. (**K**–**M**) ROC curves of a four-gene signature for distinguishing breast cancer subtypes.

To characterize the functions of the 58 candidate genes, we performed Gene Ontology (GO) and Kyoto Encyclopedia of Genes and Genomes (KEGG) pathway enrichment analyses. KEGG analysis revealed significant enrichment in pathways including chemical carcinogenesis–receptor activation and the AGE–RAGE signaling pathway in diabetic complications (Figure 2A). GO analysis revealed significant enrichment of biological process (BP) terms, including epithelial cell proliferation and response to nutrient. Enriched cellular component (CC) terms included membrane microdomain and plasma membrane raft, whereas enriched molecular function (MF) terms included growth factor binding and nuclear receptor activity (Figure 2B). To refine the candidate gene set, we applied least absolute shrinkage and selection operator (LASSO) regression analysis, which identified 23 characterized genes (Figure 2C,D) (Supplementary Table S4). The target genes were significantly differentially expressed in BRCA and controls, as shown in Figure 2E, and a significant correlation between the target genes was found by Pearson correlation analysis (Figure 2E,F). Correlation-based screening using |r| > 0.70 and *p* < 0.05 identified four genes—CAV1, CES1, PPARG, and RUNX1T1—with AUC values of 0.988, 0.948, 0.951, and 0.947, respectively (Figure 2G–J). With the correlation criteria held constant, all four genes were retained at AUC thresholds of >0.70, >0.75, and >0.80 and were selected for subsequent analyses (Supplementary Table S5). Subtype-stratified ROC analyses further showed that all four genes distinguished Luminal, HER2+, and TNBC samples from the same normal controls, with AUC values ranging from 0.943 to 0.998 in the analyzed dataset (Figure 2K–M).

### 2.3. Immune Infiltration Landscape in BRCA

**Figure 3:**
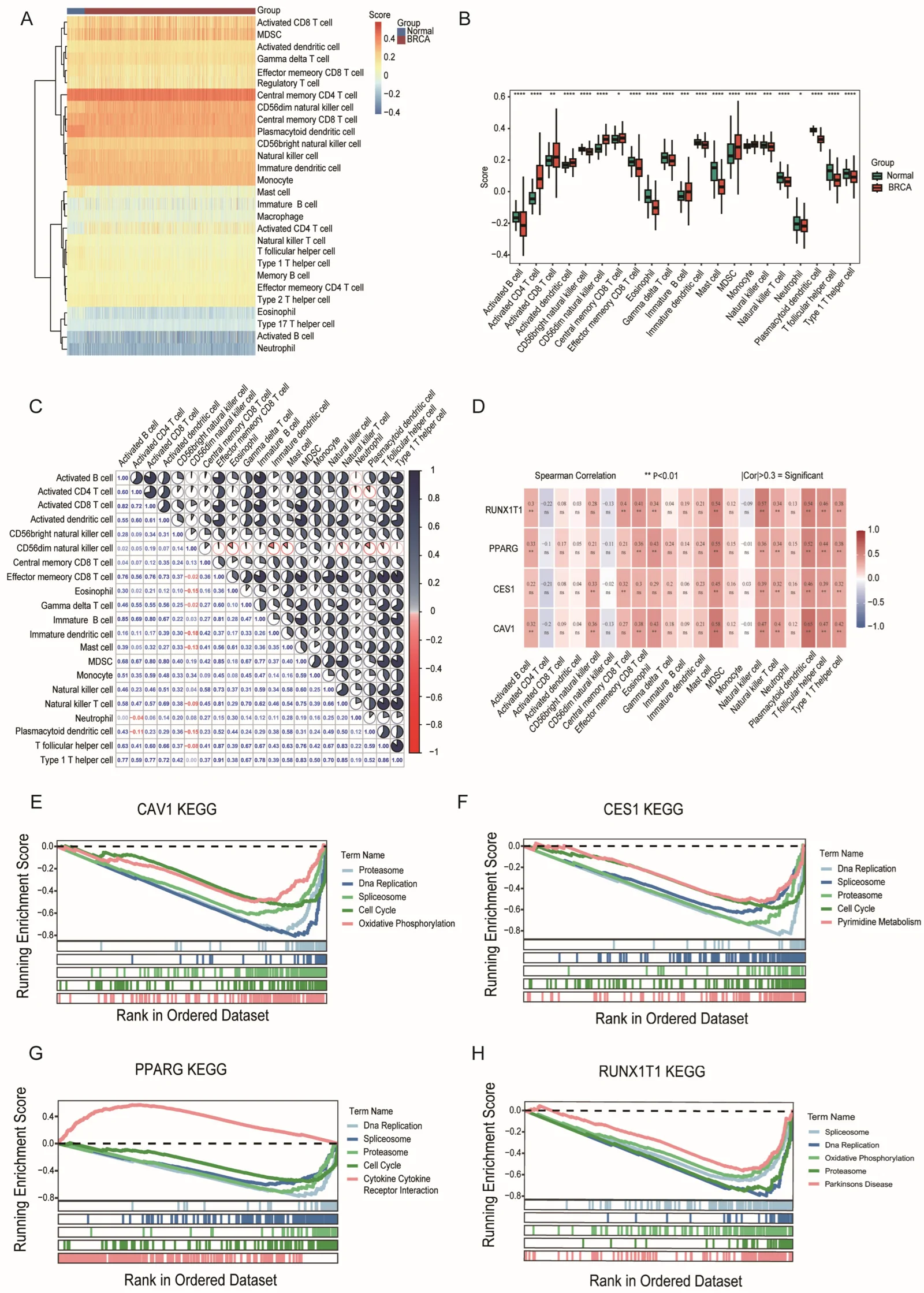
Immune infiltration analysis of BRCA. (**A**) Heatmap depicting the abundance of 28 immune cell types in BRCA and control samples. Rows represent immune cell types, and columns represent samples. (**B**) Boxplot illustrates differences in immune cell infiltration between BRCA and controls (* *p* < 0.05, ** *p* < 0.01, *** *p*< 0.001, **** *p* < 0.0001). (**C**) The correlation matrix of 28 immune cells was analyzed using Spearman’s correlation. (**D**) Correlation between target genes and immune cells. (**E**–**H**) KEGG GSEA pathway enrichment analysis of CAV1, CES1, PPARG, and RUNX1T1.

The tumor immune microenvironment plays a critical role in tumor development and metastasis [12]. To further elucidate the immune-related mechanisms associated with these key genes in BRCA, we performed immune infiltration analysis on the TCGA-BRCA dataset using the ssGSEA algorithm and then investigated the correlation between the key genes and 28 types of immune cells. Figure 3A presents a heatmap illustrating the abundance of 28 immune cells per sample in control and BRCA groups. Significant differences were observed in the proportions of 21 immune cell types between the control and BRCA group, such as central activated B cells, activated CD4 T cells, activated CD8 T cells, activated dendritic cells, and natural killer cells (Figure 3B). These findings indicated that BRCA is a highly immunogenic tumor type. Spearman’s correlation analysis revealed extensive positive correlations among multiple immune cell subsets, including activated B cells, activated CD4 T cells, activated CD8 T cells, immature B cells, gamma delta T cells, natural killer T cells, natural killer cells, and regulatory T cells (Figure 3C). The four key genes (RUNX1T1, PPARG, CES1, and CAV1) were significantly correlated with ssGSEA enrichment scores for multiple immune cell types, suggesting associations with immune-related expression patterns in BRCA (Figure 3D). Additional ssGSEA analysis using an alternative immune-cell signature collection supported several findings from the initial analysis. In both analyses, BRCA samples showed lower enrichment scores for mast cells, natural killer cells, and plasmacytoid dendritic cells than controls. CAV1, CES1, PPARG, and RUNX1T1 were positively correlated with the scores of these three cell populations in both analyses (Supplementary Figure S1C,D). GSEA revealed that CAV1, CES1, PPARG, and RUNX1T1 were significantly enriched in pathways related to cell cycle, DNA replication, oxidative phosphorylation, proteasome, spliceosome, and cytokine–cytokine receptor interaction, suggesting their potential roles in BRCA progression and immune regulation (Figure 3E–H).

### 2.4. Single-Cell Landscape and Intercellular Communication in Breast Cancer

**Figure 4:**
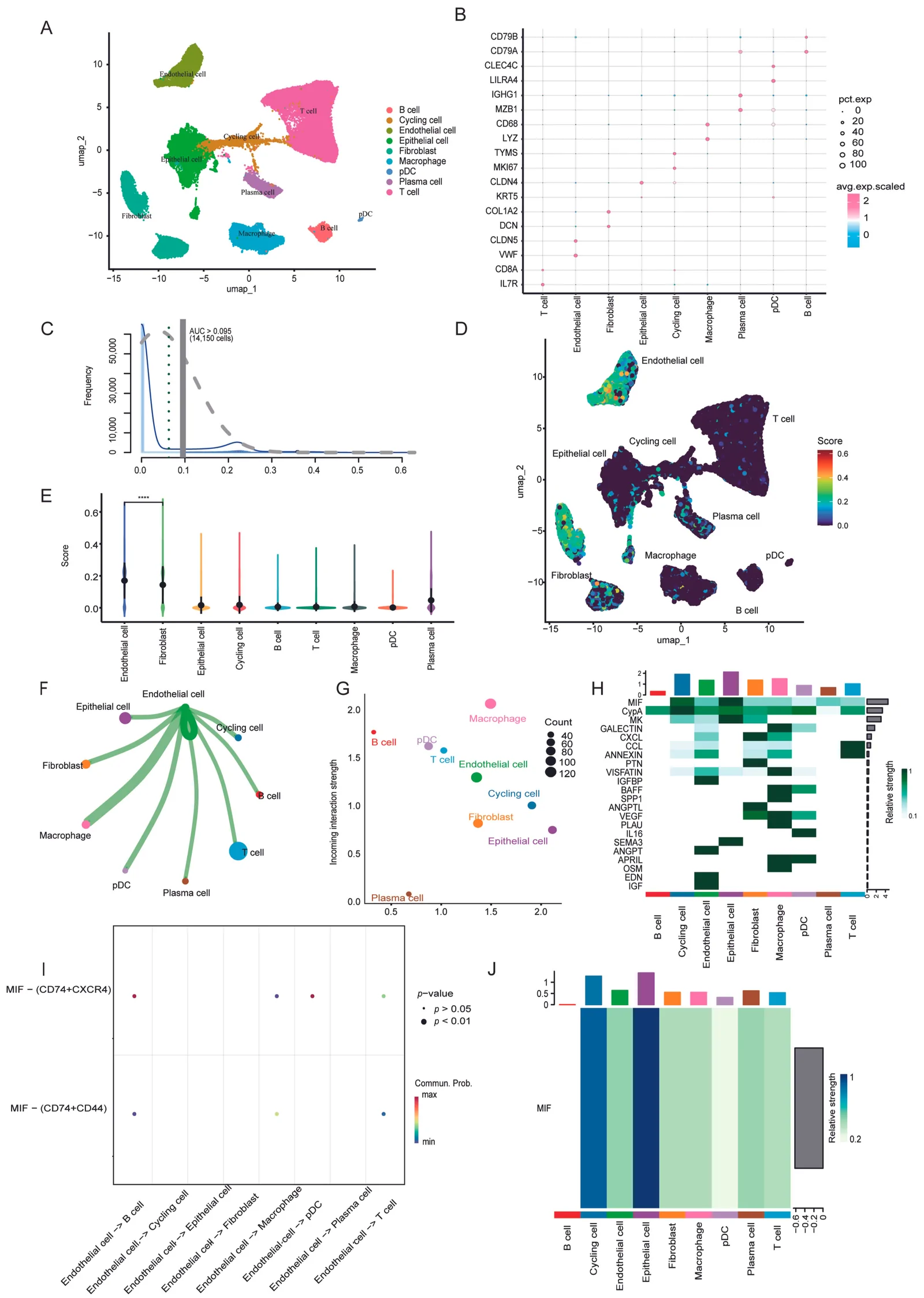
Single-Cell Transcriptomic Analysis of BRCA. (**A**) UMAP plot showing clusters of BRCA patients based on scRNA-seq. Cells are color-coded by their annotated cell types. (**B**) Bubble plots displaying the expression levels of key marker genes for different cell types. (**C**) AUC histogram of the key gene set based on AUCell analysis. (**D**) UMAP plot showing the distribution of signature scores across all cells. (**E**) Comparison of signature scores among different cell types (**** *p* < 0.0001). (**G**) Scatter plot showing the outgoing and incoming interaction strength across all pathways. (**H**) Heatmap of outgoing signaling patterns across different cell types. (**I**) Ligand–receptor interactions inferred by CellChat. (**F**) Cell–cell interaction network graph. (**J**) Bar plot showing the relative strength of MIF signaling among different cell types.

In this study, we analyzed BRCA scRNA-seq samples. After removing doublets, low-quality cells, and batch effects, a total of 83,150 high-quality cells were retained (Supplementary Figure S1E,F). Harmony integration was then performed using sample identity as the batch variable to mitigate batch effects. Uniform Manifold Approximation and Projection (UMAP) visualizations colored by sample origin before and after integration showed increased sample mixing in several regions, providing a qualitative assessment of the integration results (Supplementary Figure S1G). The cells were clustered into 22 distinct groups at a resolution of 0.5 using UMAP (Supplementary Figure S2A,B). Then, by annotating these subclusters, we identified 9 major cell types—epithelial cells (clusters 2, 11, 12, 15, 17, 18, 21), B cells (cluster 9), T cells (clusters 0, 1, 4, 14, 16), endothelial cells (clusters 5, 13), Macrophages (clusters 3, 20), Fibroblasts (clusters 6, 7), pDCs (cluster 19), cycling cells (cluster 8), and plasma cells (cluster 10)—while the remaining cells were defined as “Other” (Figure 4A). Figure 4B shows the important marker genes for each cell type. Subsequently, we calculated the scores of the 4 key genes on the upper side of scRNA-seq by AUcell (Figure 4C–E). The AUC histogram showed a clear bimodal distribution, and cells with an AUC score greater than 0.095 were considered as signature-positive cells, comprising 14,150 cells in total (Figure 4C). The four-gene signature showed the highest AUCell scores in endothelial cells (Figure 4D,E). No significant difference in endothelial or stromal cell yields was observed across ER+, HER2+, and TNBC subtypes (Kruskal–Wallis *p* > 0.05; Supplementary Figure S2D).

To further investigate the cell–cell communication networks within the BRCA tumor microenvironment, we performed cell–cell communication analysis using CellChat (version 2.1.2) (Figure 4F–J) (Supplementary Figure S2E,F). As shown in Figure 4F, endothelial cells exhibited extensive outgoing and incoming communication with multiple cell types, with the strongest interaction observed between endothelial cells and macrophages. Notably, the interaction between endothelial cells and macrophages was identified as one of the strongest communication pairs across all pathways (Figure 4G). At the pathway level, endothelial cells displayed the highest outgoing signaling strength across multiple pathways, including CXCL and VEGF, etc. (Figure 4H). Furthermore, we examined the specific ligand–receptor interactions between endothelial cells and other cell types (Figure 4I). The CD74–CXCR4 and CD74–CD44 interaction pairs were significantly enriched between endothelial cells and both macrophages and B cells, suggesting that they may play a critical role in mediating endothelial–immune cell crosstalk in the BRCA microenvironment (Figure 4I). Outgoing signaling pattern analysis of the MIF pathway revealed that endothelial cells exhibited the highest MIF signaling strength, indicating that they are a major source of MIF in the BRCA microenvironment (Figure 4J).

### 2.5. Key Genes and Regulon Landscapes of Endothelial Cell Subpopulations

To explore the predicted effects of the key genes on endothelial cell regulatory networks, we performed in virtual knockout analysis on the endothelial cell. CD74 and ACKR1 were among the genes predicted to be perturbed following simulated knockout of CAV1, CES1, or RUNX1T1. CD74 is the receptor for macrophage migration inhibitory factor (MIF) on the surface of macrophages [13,14]. Cell–cell communication analysis revealed that the interaction between endothelial cells and macrophages was the most frequent and was mediated through CD74, with high activity of the MIF signaling pathway. These findings suggest that the key genes CAV1, CES1, and RUNX1T1 in endothelial cells may regulate CD74 expression and thereby influence endothelial–macrophage crosstalk through the MIF signaling pathway, in which CD74–CXCR4 and CD74–CD44 are the major ligand–receptor pairs. Notably, the other three key genes were highly correlated with CAV1, suggesting that they may influence disease progression by regulating CAV1 and subsequently affecting CD74.

**Figure 5:**
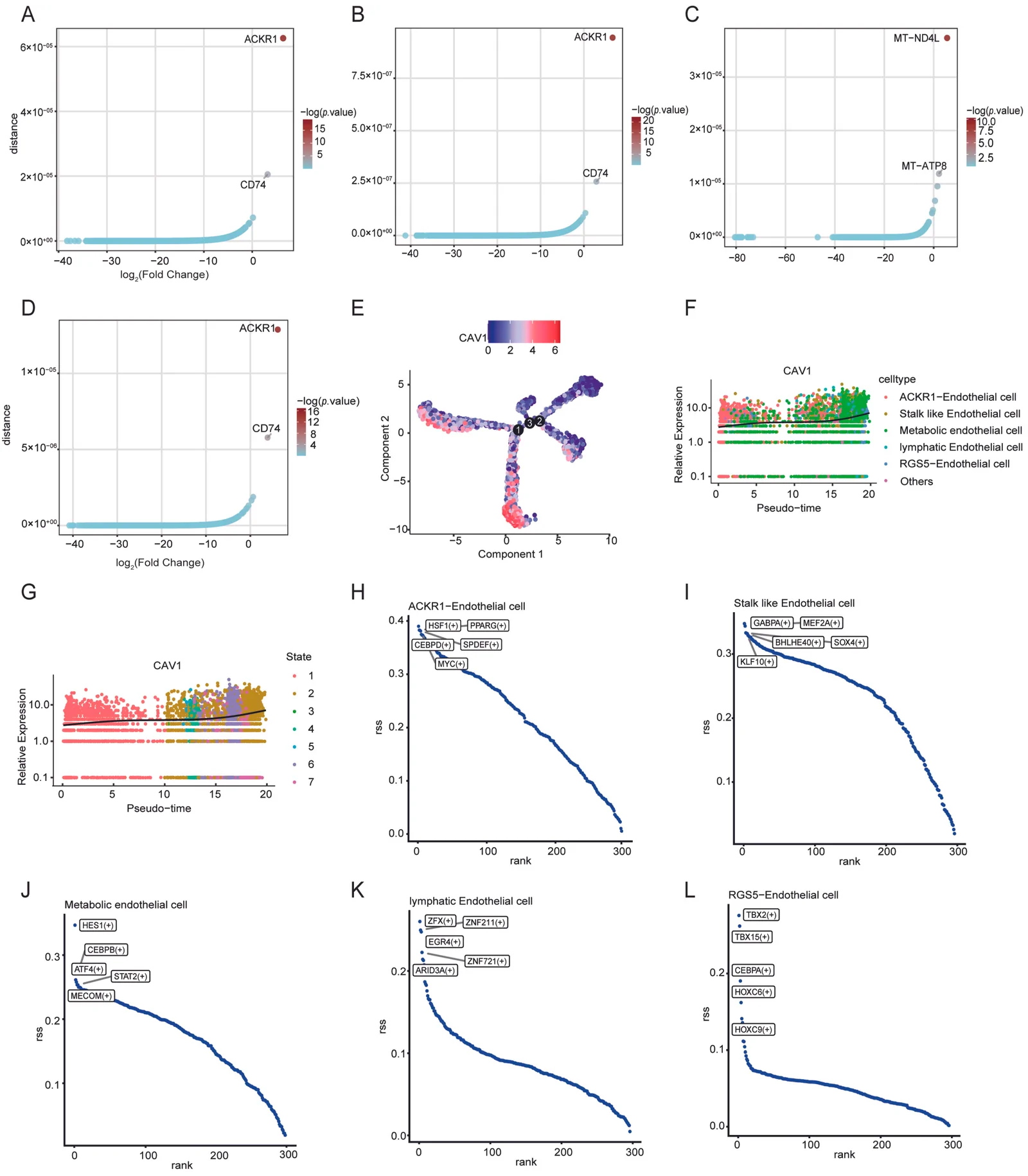
Key gene analysis. (**A**–**D**) Volcano plots showing the top affected genes after in virtual knockout of CAV1 (**A**), CES1 (**B**), PPARG (**C**), and RUNX1T1 (**D**) in endothelial cells. (**E**) Pseudotime trajectory of endothelial cell subpopulations colored by CAV1 expression. The numbers 1, 2, and 3 indicate distinct branch points along the differentiation trajectory. (**F**,**G**) Expression dynamics of CAV1 along pseudotime across the five endothelial cell subpopulations and the seven cell states. (**H**–**L**) Regulon specificity score (RSS) ranking plots showing the top transcription factors enriched in each endothelial cell subpopulation: ACKR1-Endothelial cells (**H**), Stalk-like endothelial cells (**I**), metabolic endothelial cells (**J**), lymphatic endothelial cells (**K**), and RGS5-Endothelial cells (**L**).

To investigate the expression dynamics of CAV1 across endothelial cell subpopulations, we performed pseudotime trajectory analysis. Based on the expression of marker genes, five endothelial cell subpopulations were identified: ACKR1-Endothelial cells, Stalk-like endothelial cells, metabolic endothelial cells, lymphatic endothelial cells, and RGS5-Endothelial cells [15,16] (Supplementary Figure S2C). As shown in Figure 5E, CAV1 was dynamically expressed along the trajectory, with high expression observed at specific branch points. The expression of CAV1 varied among the five endothelial cell subpopulations along pseudotime (Figure 5F). Specifically, CAV1 expression was relatively higher in ACKR1-Endothelial cells and metabolic endothelial cells, while it remained low in lymphatic endothelial cells and RGS5-Endothelial cells. Similar trends were observed when cells were grouped by state (Figure 5G).

To identify the key transcriptional regulators in each endothelial cell subpopulation, we performed regulon specificity score (RSS) analysis (Figure 5H–L). The top enriched transcription factors were as follows: HSF1 (+), PPARG (+), and MYC (+) in ACKR1-Endothelial cells (Figure 5H); GABPA(+), MEF2A(+), and KLF10(+) in Stalk-like endothelial cells (Figure 5I); HES1(+), CEBPB(+), and HECOM(+) in metabolic endothelial cells (Figure 5J); ZFX(+), ZNF211(+), and ARID3A(+) in lymphatic endothelial cells (Figure 5K); and TBX2(+), TBX15(+), and HOXC9(+) in RGS5-Endothelial cells (Figure 5L).

## 3. Discussion

Breast cancer (BRCA) remains one of the most common malignancies and a leading cause of cancer-related mortality among women worldwide. In this study, we integrated transcriptomic and scRNA-seq analyses to characterize key genes and cellular crosstalk underlying BRCA progression, with a particular focus on CAV1 and endothelial–macrophage communication.

Through differential expression analysis and WGCNA, we identified 1261 genes significantly correlated with BRCA development. Combined with disease-associated genes from public databases, 58 candidate genes were screened and further refined by LASSO regression. Among the 23 characterized genes, CAV1, CES1, PPARG, and RUNX1T1 exhibited potential discriminatory value. Notably, CAV1 had the highest observed AUC among these four genes. CAV1 encodes caveolin-1, a structural protein of caveolae that regulates multiple signaling pathways involved in cell proliferation, migration, and tumor progression [17,18]. Emerging evidence has implicated CAV1 in the regulation of the tumor immune microenvironment, particularly through its influence on endothelial cell function and immune cell recruitment [19].

Immune infiltration analysis revealed that the four key genes were significantly correlated with multiple immune cell types. GSEA further demonstrated that these genes were enriched in pathways related to cell cycle, DNA replication, oxidative phosphorylation, and cytokine–cytokine receptor interaction, indicating their involvement in both tumor progression and immune regulation.

Single-cell analysis revealed that the four key genes were most highly enriched in endothelial cells, and CellChat inferred MIF-related communication between endothelial cells and macrophages. CD74 is the receptor for MIF on the surface of macrophages [20]. Virtual knockout analysis identified CD74 among the genes predicted to be perturbed following CAV1, CES1, or RUNX1T1 knockout in endothelial cells.

Pseudotime trajectory analysis further revealed that CAV1 was dynamically expressed across endothelial cell subpopulations, with relatively higher expression in ACKR1-Endothelial cells and metabolic endothelial cells. Regulon specificity score analysis identified distinct transcriptional regulatory networks in each subpopulation, including PPARG and MYC in ACKR1-Endothelial cells, and TBX2 and CEBPA in RGS5-Endothelial cells. These findings highlight the heterogeneity of endothelial cells in BRCA and suggest subpopulation-associated variation in CAV1 expression. These findings suggest that CAV1 may participate in BRCA progression by influencing the functional states of endothelial cell subpopulations. MIF–CD74 signaling plays a prominent role in breast cancer progression, and MIF inhibition can convert “cold” tumors into “hot” tumors and enhance immune checkpoint blockade efficacy. Given its multiple protumor functions, MIF has emerged as a promising therapeutic target [21].

This study has several limitations. First, it is based on the reanalysis of publicly available datasets; however, the datasets included in this study have a sufficient sample size, which supports the reliability of the findings and provides important guidance for future research. Second, although the virtual knockout [22,23] and cell–cell communication results are computational predictions, they were generated using a well-established and reliable bioinformatics pipeline, which offers meaningful guidance for subsequent experimental studies. Third, strict thresholds were applied when selecting target genes corresponding to drug compounds, further supporting the reliability of the prioritized candidates. Finally, the discriminatory performance of the candidate genes requires validation in independent cohorts before clinical applicability can be assessed.

In summary, our study identifies CAV1 as a potential gene in BRCA, regulating endothelial–macrophage crosstalk through the CD74/MIF pathway. These findings provide new insights into the molecular mechanisms of BRCA progression and suggest that targeting the CAV1–CD74 regulatory interaction may represent a novel therapeutic strategy for BRCA.

## 4. Materials and Methods

### 4.1. Data Acquisition

We obtained the breast cancer dataset from The Cancer Genome Atlas (TCGA) through USCS database, which included 1109 BRCA and 113 normal tissue samples. After filtering, 1084 primary tumor samples and 113 normal tissue samples were retained for subsequent analysis. Additionally, single-cell RNA-seq data for BRCA were downloaded from Gene Expression Omnibus (GEO) database (http://www.ncbi.nlm.nih.gov/geo/ (accessed on 30 January 2025)), accession number GSE176078, including 26 primary tumor samples (11 ER+, 5HER2+ and 10 TNBC) [24]. All subtypes were retained for downstream analysis. To assess whether stromal and endothelial cell yields differed across clinical subtypes, we compared the proportions of these cell types among ER+, HER2+ and TNBC subsets using the Kruskal–Wallis test.

### 4.2. Candidate Gene Curation and Prioritization

The Traditional Chinese Medicine Systems Pharmacology Database and Analysis Platform (TCMSP) was used to retrieve genes associated with the main active ingredients of Fufang Banmao Capsules. Active compounds from Fufang Banmao Capsules were retrieved from the TCMSP database and screened using the standard pharmacokinetic thresholds of oral bioavailability (OB) ≥ 30% and drug-likeness (DL) ≥ 0.18. Specifically, we identified 4 *Banmao*-associated genes, 13 genes related to *Curcuma zedoaria*, 43 genes related to *Sangleng*, 7 genes related to *Macrocarpium officinale*, and 143 genes related to *Glycyrrhiza*. By integrating these datasets, a total of 148 drug genes were obtained (Supplementary Tables S6 and S7).

BRCA-related genes were retrieved from the GeneCards [25] and Online Mendelian Inheritance in Man (OMIM) [26] databases using the keyword “breast cancer”. To explore gene co-expression patterns, we applied the WGCNA [27] package in R. Before network construction, samples were hierarchically clustered based on their gene expression profiles using Euclidean distance and complete linkage with the hclust function. The resulting dendrogram was cut at a height of 105 using cutreeStatic, with a minimum cluster size of 10. Only samples assigned to the largest cluster were retained for subsequent analysis. The soft thresholding power β-value was screened during module construction by the pick-Soft-Threshold function in WGCNA to ensure the construction of scale-free network. The module Eigengenes function was used to calculate the relationship between module-characterized genes and BRCA and control samples, identifying potential disease-related gene modules. Then, the genes enriched in the hub module were intersected with the identified DEGs.

Subsequently, the intersection of drug-related genes, disease-related genes and DEGs were performed. To get more precise pathogenic genes highly related to BRCA, the overlapping genes were further selected by performing LASSO algorithm through glmnet [28] R package. ROC curves of these identified characteristic genes were generated by pROC [29] R package.

### 4.3. Immune Infiltration and Correlation Analyses

Immune cell infiltration in the TCGA-BRCA cohort was assessed using ssGSEA implemented in the GSVA R package [30]. Two published immune cell signature collections were analyzed separately: the signatures for 28 immune cell types defined by Charoentong et al. [31] and those for 24 immune cell types used by Şenbabaoğlu et al. [32]. For each sample, ssGSEA enrichment scores were calculated for each signature and used as estimates of relative immune cell infiltration. Heatmaps, box plots, and Pearson correlation analyses were then performed to visualize infiltration patterns, compare abundances between BRCA and control groups, and assess the correlations among immune cell types and between the key genes and immune cell subsets.

### 4.4. Single-Cell RNA-Seq Data Processing and Cell Annotation

ScRNA-seq data were processed and analyzed using the Seurat [33] package. The CreateSeuratObject function was used to generate the Seurat object. The DoubletFinder package was used to remove doublets, of which 7.5% were doublets. In addition, the percentage of mitochondrial genes was calculated using the PercentageFeatureSet function, and cells with percent.mt ≥20% were excluded. Low-quality cells were removed using the subset function with the following criteria: nCount_RNA > 100 and <17,000, and nFeature_RNA > 200. All samples were integrated into a Seurat object using the merge function, and the harmony package was utilized to remove batch effects. Clustering was performed using the FindClusters function with a resolution range of 0.1–1, and the optimal resolution was selected based on the clustering stability and biological interpretability of the resulting clusters. To analyze the characteristics of each Cell cluster, the DEGs for each cluster were identified by the “FindAllMarkers” function (min.pct = 0.25, logfc. threshold = 0.25, *p* < 0.05). Marker genes were obtained by screening the literature and searching the CellMarker 2.0 database [34] (http://bio-bigdata.hrbmu.edu.cn/CellMarker/ (accessed on 30 July 2026)).

### 4.5. Gene Signature Scoring and Cell–Cell Communication Analysis

The expression of 4 key genes in BRCA scRNA-seq data was evaluated using the AUcell [35] package and the activity scores for each cell cluster sample were visualized by ggboxplot and combined with UMAP dimensionality reduction plot to clearly show the distribution of gene set activity in different cell clusters. To explore the potential cell–cell communication, the ligand–receptor interactions between different cells were analyzed using the R package CellChat (version 2.1.2) [36].

### 4.6. Endothelial Cell Heterogeneity and in Silico Perturbation Analysis

Gene regulatory networks were inferred using pySCENIC [37] with the GRNBoost2 algorithm, and regulon activity was quantified per cell using AUCell. Pseudotime trajectories were reconstructed using Monocle2 [38], and gene expression dynamics along the trajectory were visualized with the plot cell’s function. In silico gene knockout was performed using scTenifoldKnk (version 1.1) [39] to predict the regulatory effects of key genes on endothelial cell identity.

### 4.7. Functional Enrichment and Gene Set Enrichment Analyses

Gene symbols were converted to Entrez IDs using the org.Hs.eg.db package, followed by GO and KEGG pathway enrichment analysis through the ClusterProfiler [40] package (*p* < 0.05). KEGG results are visualized by the “barplot” function, and GO results are visualized by the “dotplot” function. Spearman’s correlation coefficients between each key gene and all protein-coding genes were calculated using the TCGA-BRCA dataset, and genes were ranked in descending order of correlation. GSEA was then performed using the clusterProfiler [40] package with the KEGG gene sets from MSigDB [41], and significantly enriched pathways were defined as |NES| > 1, *p* < 0.05, and q < 0.25.

## 5. Conclusions

In summary, our integrative bioinformatics analysis identified CAV1, CES1, PPARG, and RUNX1T1 as candidate BRCA-associated genes with potential discriminatory value in the analyzed data. Single-cell analysis suggested preferential expression of these genes in endothelial cells and inferred CD74–CXCR4 and CD74–CD44 mediated communication between endothelial cells and macrophages. Virtual knockout analysis predicted changes involving CD74 following simulated knockout of CAV1, CES1, or RUNX1T1 in endothelial cells. Together, these computational findings generate hypotheses regarding endothelial–macrophage interactions and a possible relationship between CAV1 and CD74 in BRCA.

## Data Availability

Publicly available datasets were reanalyzed in this study. Bulk transcriptomic data from The Cancer Genome Atlas breast invasive carcinoma project (TCGA-BRCA) were downloaded from the UCSC Xena platform (https://xena.ucsc.edu/(accessed on 15 January 2025)). Single-cell RNA-sequencing data were obtained from the Gene Expression Omnibus (GEO) under accession number GSE176078 (https://www.ncbi.nlm.nih.gov/geo/query/acc.cgi?acc=GSE176078 (accessed on 30 January 2025)).

## Supplementary Materials

The following supporting information can be downloaded at https://www.mdpi.com/article/10.3390/ijms27209298/s1.

## Abbreviations

BRCA	breast cancer
GO	Gene Ontology
KEGG	Kyoto Encyclopedia of Genes and Genomes
BP	biological process
CC	cellular component
MF	molecular function
PPI	protein–protein interaction
WGCNA	weighted gene co-expression network analysis
TF	transcription factor
LASSO	least absolute shrinkage and selection operator
ROC	receiver operating characteristic
AUC	area under the curve
DEGs	differentially expressed genes
ssGSEA	single-sample gene set enrichment analysis
